# Increased Cervical CD4^+^CCR5^+^ T Cells Among Kenyan Sex Working Women Using Depot Medroxyprogesterone Acetate

**DOI:** 10.1089/aid.2018.0188

**Published:** 2019-02-28

**Authors:** Julie Lajoie, Annelie Tjernlund, Kenneth Omollo, Gabriella Edfeldt, Maria Röhl, Geneviève Boily-Larouche, Julianna Cheruiyot, Makubo Kimani, Joshua Kimani, Julius Oyugi, Kristina Broliden, Keith R. Fowke

**Affiliations:** ^1^ Department of Medical Microbiology and Infectious Diseases, University of Manitoba, Winnipeg, Canada.; ^2^ Department Medical Microbiology, University of Nairobi, Nairobi, Kenya.; ^3^ Department of Medicine Solna, Center for Molecular Medicine, Karolinska Institutet, Karolinska University Hospital Solna, Stockholm, Sweden.; ^4^ Partners for Health and Development in Africa, Nairobi, Kenya.; ^5^ Department of Community Health Science, University of Manitoba, Winnipeg, Canada.

**Keywords:** depot medroxyprogesterone acetate (DMPA), HIV target cells, immune activation, ectocervix, cervical biopsy, contraception

## Abstract

Depot medroxyprogesterone acetate (DMPA) is the most common hormonal contraceptive used by women in sub-Saharan Africa, however, it has been epidemiologically associated with HIV infections. To assess whether DMPA has an effect on the number and activation of HIV target cells, this study assessed the levels and phenotype of blood- and mucosal-derived HIV target cells among women using DMPA. Thirty-five HIV uninfected women from the Pumwani Sex Worker cohort from Nairobi, Kenya were enrolled in the study (15 using DMPA and 20 not using hormonal contraception). Blood (plasma and peripheral blood mononuclear cells) and cervicovaginal (lavage, cervical cells, and ectocervical biopsies) samples were collected. Cellular phenotype and activation status were determined by flow cytometry, cytokine levels were assessed by bead array and image analysis assessed cell number and phenotype *in situ.* In blood, the proportion of HIV target cells and activated T cells was lower in DMPA users versus those not using hormonal contraceptives. However, analysis of cervical mononuclear cells showed that DMPA users had elevated levels of activated T cells (CD4^+^CD69^+^) and expressed lower levels of the HIV co-receptor CCR5 on a per cell basis, while tissue samples showed that in the ectocervix, DMPA users had a higher proportion of CD4^+^CCR5^+^ T cells. This study demonstrates that DMPA users had higher levels of activated T cells and HIV target cells in the genital tract. The increased pool of mucosal HIV target cells provides new biological information about the potential impact of DMPA on HIV susceptibility.

## Introduction

Access to hormonal contraception (HC) enables women to make choices about their reproductive health. Long-term hormonal contraceptives such as injectable contraceptives are common, particularly in younger women, mostly due to their ease of use.

Depot medroxyprogesterone acetate (DMPA) is an intramuscular injectable progestin that is administrated once every 3 months. DMPA is the most common HC used in sub-Saharan Africa in women between the ages of 18–49 years.^1^ Simultaneously, this group is significantly overrepresented in the number of new HIV infections.^2^ Recently, meta-analyses have reported that DMPA use was associated with about 1.4-fold increase in risk of HIV infection.^3–5^ Understanding how DMPA might affect the susceptibility to HIV infection at the cellular and molecular level is essential for the development of new policies on its use.

Many biological mechanisms have been suggested to explain the increase in HIV risk associated with DMPA use, such as changes in the vaginal microbiome,^6^ slight thinning of the vaginal epithelium,^7^ decrease of epithelial repair proteins,^8^ increased mucosal epithelial permeability,^9^ changes in inflammatory cytokine production,^10,11^ and changes in numbers of HIV-susceptible T cells in the vaginal tissue.^6,12,13^ A study conducted in rhesus macaques showed that DMPA-treated animals expressed higher levels of α4β7 on endocervical CD4^+^ T cells,^14^ a phenotype that is highly susceptible to HIV infection.^15^ Finally, an *in vitro* study showed that MPA (medroxyprogesterone acetate) treatment prevents the downregulation of CCR5 and increases HIV replication in activated peripheral blood mononuclear cells (PBMC).^16^

Analysis of the upper reproductive tract from women on DMPA showed recruitment of macrophages and increased proportions of activated CD4^+^ and CD8^+^ T cells to the endometrium AND decreased levels of interleukin (IL)-1β and IL-6 in the endocervix. However, no increase in the CCR5 expression on CD4^+^ T cells in either the endocervix or endometrium was seen when compared with women not using HC.^17^

Cells other than CD4^+^ T cells can also facilitate HIV infection. Langerhans cells (LCs) are a subset of dendritic cells (DCs) that line mucosal epithelia and can sense and induce the immune system to fight invading pathogens. In the genital epithelium, they appear to have conflicting functions in terms of HIV pathogenesis: they are one of the primary targets of HIV infection,^18^ while also forming a protective barrier against infection and transmission by capture of HIV through the C-type lectin langerin, leading to degradation of the virus in the Birbeck granules, which are characteristic of Langerhan cells.^19,20^

Interestingly, it was recently proposed that restriction by human TRIM5alpha is controlled by C-type lectin receptor-dependent uptake of HIV, dictating protection, or infection, of human DC subsets.^21^ It has also been proposed that genital Langerin^+^CD1a^+^ cells do not harbor Birbeck granules and may, therefore, retain virus more easily.^22^

The impact of DMPA usage on women who are at high risk of HIV infection, such as female sex workers (FSWs), remains mostly unknown. We have previously shown that FSWs display an altered level of immune activation compared with women from the general population.^23^ This highlights the importance of understanding how the use of DMPA impacts the immune environment and HIV susceptibility in these women at high risk of infection. The purpose of this study was to compare the blood and cervicovaginal levels of HIV target cells and immune activation in women involved in sex work who were DMPA users versus a matched group that did not use HC.

## Materials and Methods

### Participants and study design

This cross-sectional study involved HIV-seronegative women from the Pumwani Sex Worker Cohort, Nairobi, Kenya. Participants who were selected were involved in sex work for 3 years or less. The case group were women using DMPA as hormonal contraceptive (*n* = 15) and the control group consisted of women from the same cohort not using any form of hormonal contraception (no HC, *n* = 20). The control women were all sampled in the luteal phase of their menstrual cycle, which is associated with high progesterone levels and thus most closely matches the conditions of women using DMPA. Written informed consent was obtained from all participants' prior enrolment in the study. The Ethical Review Boards from the University of Manitoba, the Kenyatta National Hospital/University of Nairobi and the Regional Ethical Review Board in Stockholm approved the study.

### Enrolment and clinical procedures

Exclusion and inclusion criteria have been described previously.^24^ Briefly, inclusion criteria at enrolment were as follows: age between 18 and 50; not being menopausal; no prior hysterectomy; not pregnant or breastfeeding and negative for HIV, *Neisseria gonorrhoeae* (NG), *Chlamydia trachomatis* (CT), or syphilis infection. To be enrolled in this study, participants had to self-declare as sex workers and be involved in sex work for 3 years or less.

Participants on DMPA had to be on this family planning method for at least 6 months and had their last DMPA injection 2–6 weeks before first visit in the study. The samples collected in this study were 2 weeks later (4–8 weeks post DMPA injection). For the control group, the phase of the menstrual cycle was defined by date since last onset of menses (day 21, interquartile range 19.8–24.0) and measurement of progesterone concentration in plasma using the MILLIPLEX MAP Steroid/Thyroid Hormone Magnetic Bead Panel (Millipore, Merck, Darmstadt, Germany). Women were only included in these analyses if the progesterone ratio was 2.0 or greater when comparing the study sample with a sample collected 2 weeks earlier.

Participants enrolled in the study answered a demographic and behavioral questionnaire. HIV serology using rapid test (Determine, Inverness Medical, Japan) was performed at screening and again 8 weeks following sample collection. Bacterial vaginosis (BV) was defined using the Nugent's score. Presence of *Trichomonas vaginalis* was diagnosed using normal saline microscopy. Urine samples were collected for PCR detection of NG and CT (Roche Amplicor kits).

### Sample collection and processing

Peripheral blood samples, cervical mononuclear cells (CMCs), cervico-vaginal lavage (CVL) samples, and ectocervical tissue biopsies were obtained from all participants. The blood samples were collected by venipuncture using a sodium heparin tube and PBMC were isolated using a ficoll density gradient. The CVLs and CMCs collection methods have been reported previously.^25^ Briefly, CVLs were collected using 2 mL of sterile phosphate-buffered saline (PBS) flushed into the vaginal cavity and collected from the posterior fornix region. Samples were kept on wet ice and transported to the laboratory where they were centrifuged to remove cell debris and the lavage supernatants were stored at −80°C.

CMCs were obtained by scraping the ectocervix with a cervical spatula and the endocervix with a cervical brush. The cytobrush and cervical spatula were transferred into sterile PBS, kept on ice until being processed in the laboratory. Ectocervical tissue samples (sometimes including also a small part of the endocervix) were collected using a Schubert biopsy forceps (model ER058R Aesculap, Germany) by a trained gynecologist, and consisted of a 3 mm^3^ biopsy from the superior portion of the ectocervix. Immediately after sampling, the biopsy was snap frozen in liquid nitrogen and subsequently stored at −80°C. Ferric subsulfate solution gel (20%–22% ferric subsulfate, Monsel solution; Gynex, WA) was applied to the wound to prevent bleeding. The gynecologist ensured that only minor bleeding was visible before the participant left the clinic. Participants returned 3 days later to the clinic and the gynecologist inspected their cervix to ensure healing. Participants were counseled to not have unprotected sex for 2 weeks after the biopsy to allow proper healing.^24^

### Flow cytometry

PBMC (10^6^ cells/donor) and CMC were stained for *ex vivo* cellular phenotype using CD3-PE.Cy5 (clone UCHT1; BD Biosciences), CD4-FITC (clone RPA-T4; BD Biosciences), and CD161-APC (clone DX12; BD Biosciences). The cells were also stained for the HIV co-receptor CCR5-V450 (clone 2D7/CCR5; BD Biosciences), and cellular activation markers CD69-PE.Cy7 (clone FN50; BD Biosciences), CD95-PE (clone DX2; BD Biosciences), and HLA-DR-APC.H7 (clone L243; BD Biosciences). Dead cells were eliminated from the analysis using Far Red-Live/Dead discrimination (Invitrogen).

Data were acquired on an LSRII flow cytometer (BD System) and analyzed using FlowJo v10.0.8r1 (TreeStar). Samples were gated on forward scatter height (FSC-H) and forward scatter area (FSC-A) for excluding doublets, then on lymphocytes population with a side scatter area (SSC-A) and FSC-A gating. From the lymphocytes gate, live cells were gated with the far-Red-live dead/SSC-A. CD3^+^ cells were gated from the live cells and CD4^+^ T cells from the CD3^+^ gates (Supplementary Fig. S1). Cervical samples with less than 100 CD3^+^ cells were excluded from the analyses.

### Cytokines/chemokines detection

Plasma and cervico-vaginal concentrations of cytokines and chemokines were measured by Milliplex (Millipore, Merck KGaA, Darmstadt, Germany) according to the manufacturer instructions and analyzed on the BioPlex-200 (Bio-Rad, Mississauga, Canada). The following were detected: IFN-γ, IL-12-p70, sCD40L, IL-10, IL-17A, IL–1α, IL-1β, IL-2, IL-8, IL-15, TNF-α, interferon gamma-induced protein (IP)-10, monocyte chemoattractant protein (MCP)-1, macrophage inflammatory protein (MIP)-1α, MIP-1β, IL1RA, monokine induced by gamma interferon, MIP3β, and soluble (s)IL-2RA. All samples were run in duplicate and the mean was used for the analyses.

Plasma levels were measured according to the 2-hour manufacturer protocol while CVLs were incubated overnight to improve the sensitivity of the detection as previously described.^26^ Undetectable samples, with a value lower than the lower limit of detection (LLD), were assigned a value of LLD/2 and samples above the upper limit of detection (ULD) were assigned with the ULD+1000.

### *In situ* fluorescence staining

Immunofluorescent staining was performed on 8-μm thick sections of the cryopreserved tissue samples, as previously described.^27^ Briefly, the tissue sections were fixed in 2% formaldehyde, and the staining was done sequentially. The following primary monoclonal antibodies were used; rabbit anti-human CD4 antibody (clone EPR6855; Abcam, Cambridge, England), mouse anti-human CCR5 antibody (clone MC-5, kindly provided by Professor M. Mack from the University Clinic of Regensburg, Germany), and rat anti-human Langerin antibody (clone 929F3.01; Dendritics, Lyon, France). Fluorescent-labeled secondary antibodies used were Alexa Fluor 594-conjugated donkey anti-rabbit IgG antibody, Alexa Fluor 488-conjugated donkey anti-mouse IgG antibody, and Alexa Fluor 488-conjugated donkey anti-rat IgG antibody (Molecular Probes, Life Technologies Europe BV, Stockholm, Sweden). Antigen retrieval (pure methanol +0.5% hydrogen peroxide) was needed before addition of the primary CD4 antibody. Tissue sections were counterstained with DAPI (Molecular Probes, Life Technologies) to stain the nuclei, washed and thereafter mounted with Fluorescent Mounting Medium (Dako, Carpinteria, CA). Negative controls were included and consisted of incubations in the presence of secondary antibodies alone.

### Image analysis

The stained tissue sections were scanned into digital images using a Pannoramic 250 Flash Slide Scanner (3DHistech Kft., Budapest, Hungary). The epithelial compartment of the ectocervical tissue was manually outlined in regions of interest in Pannoramic Viewer (version 1.15.3; 3DHistotech Ltd., Budapest, Hungary), on all images before analysis. Computerized image analysis of the immunofluorescent double staining was performed using CellProfiler.^28^ To segment out positively stained cell area from background autofluorescence, a white tophat noise-reduction filter was used together with a fixed threshold for the CD4 and Langerin staining. For the CCR5 staining an image-dependent intensity threshold, defined as the upper quartile intensity in each image multiplied by two, was used. The frequency of positively stained cells was expressed as the percentage of positively stained area out of total tissue area, and out of positively stained CD4 tissue area. Areas with broken tissue, cysts, artefacts, or detached epithelium were excluded.

### Statistical methods

The group that was not taking hormonal contraception (no HC) was considered the control group and the group taking DMPA as hormonal contraception was considered cases. Gaussian distribution was tested by Shapiro–Wilk normality *t* test. Distribution of categorical variables was measured by Fisher's exact test. Chi-square was used to measure the distribution of BV between the two groups.

One-way analysis of variance (univariate analysis) was used for comparison of continuous variable between the two groups (Kruskal–Wallis test for not normally distributed and Mann–Whitney *U* test for normally distributed markers). Multiple linear regressions (multivariate analysis) were performed for adjusting statistical analysis for co-founding variables (“duration of sex work” and “douching”). Spearman's rank test was used for correlation between not normally distributed variables while Pearson coefficient correlation was used to determine correlations between duration on DMPA and normally distributed continuous variables.

Most cytokines and chemokines expression showed a skewed distribution and therefore were log10 transformed for further analysis. Statistical analyses were performed using GraphPad Prism 6 (version 6.0; GraphPad Software). Normality test and multiple linear regressions were performed using SPSS (version 24; IBM). All *p*-values were two-sided and values *p* ≤ .05 were considered as significant. Given the discovery approach of this study, we kept a *p*-value of ≤.05 to not miss a real effect and analyses were not corrected for multiple comparisons.

## Results

### Demographics

To investigate whether DMPA use affects the systemic and genital mucosa immunological milieu, we collected paired blood, CMC and CVL samples, and ectocervical tissue biopsies from DMPA users (*n* = 15) and no HC users (*n* = 20; controls) (Table 1). Women in the DMPA group used the high progestin injectable as contraceptive method since a median of 3 years. The corresponding control group were sampled during the progesterone-high luteal phase [day in menstrual cycle, median number: 21 days; progesterone ratio (luteal:follicular phase), median value: 6.2].

**Table 1.** Sociodemographics and Behavioral Characteristics of Each Study Group

	*DMPA,* n* = 15*	*No HC,* n* = 20*	p*-Value*
Age (years), mean (SD)	31.6 (4.5)	33.1 (7.9)	ns
Duration of sex work (months), median (IQR)	21 (12–24)	30 (24–36)	.01^a^
Duration on DMPA (years), median (IQR)	3.0 (3.0–5.0)	—	
Days since last menses, median (IQR)	—	21.0 (19.8–24.0)	
Ratio progesterone luteal:follicular, median (IQR)		6.2 (5.1–21.2)	
Number of clients in the last 7 days, mean (SD)	6.6 (6.7)	6.7 (7.8)	ns
Number of protected sex act with clients in the last 7 days, mean (SD)	6.9 (6.8)	7.0 (7.9)	ns
Having a regular partner, *n* (%)	8 (53)	14 (63.6)	ns
Practicing vaginal douching, *n* (%)	7 (46.7)	2 (9.5)	.02^b^
Presence of visual cervical inflammation, *n* (%)	1 (6.7)	2 (9)	ns
Bacterial vaginosis			ns
Normal	8	13	
Intermediate	4	4	
Positive	3	4	

Two additional women in the control group (no HC) were found to be infected with *Neisseria gonorrhoeae* and *Chlamydia trachomatis*, respectively, after enrollment and were therefore not excluded from entering the study. These two women are clearly marked, when included in the following figures.

^a^ Mann–Whitney *t* test.

^b^ Fisher's exact test.

DMPA, depot medroxyprogesterone acetate; No HC, not taking hormonal contraception; IQR, interquartile range; *n*, number; ns, non significant; SD, standard deviation.

The study groups were similar in age, number of clients, number of protected sex acts, having a regular partner, presence of visual cervical inflammation, and BV status. However, a significant difference was observed for the duration of self-reported sex work (DMPA users median of 21 vs. 30 months in the no HC group, *p* = .01) and the practice of vaginal douching (DMPA 47% vs. no HC 10%, *p* = .02). None of the women in the study seroconverted to HIV when tested 8 weeks following sample collection.

### Impact of DMPA usage on blood-derived CD4^+^ T cells

Using multi-parametric flow cytometry, we first addressed whether DMPA-usage altered the numbers of potential HIV target cells, and their activation phenotype in blood. In this analysis, 22 participants were taken in consideration. Two were diagnosed with a sexually transmitted infection after recruitment and sampling and are therefore clearly marked.

In the univariate analysis (Supplementary Fig. S2A), the DMPA users had a significantly higher percentage of CD4^+^ T cells among total blood-derived CD3^+^ T cells when compared with the no HC users (median: 70% vs. 69%, *p* = .02, respectively). However, among the CD4^+^ T cell population, the DMPA users displayed significantly lower percentage of HIV target cells (median % of CD4^+^CCR5^+^ T cells: 1% vs. 7%, *p* = .0008), including activated HIV target cells (median % CD4^+^CCR5^+^CD69^+^ T cells: 0.1% vs. 0.3%, *p* = .04) and significantly lower percentage of activated CD4^+^ T cells (early activation: median % of CD4^+^CD69^+^ T cells: 1% vs. 8%, *p* < .0001; mid-activation: CD4^+^CD95^+^ T cells: 62% vs. 63%, *p* < .05; and late activation: CD4^+^HLA-DR^+^ T cells: 1% vs. 2%, *p* = .04).

**FIG. 1.**
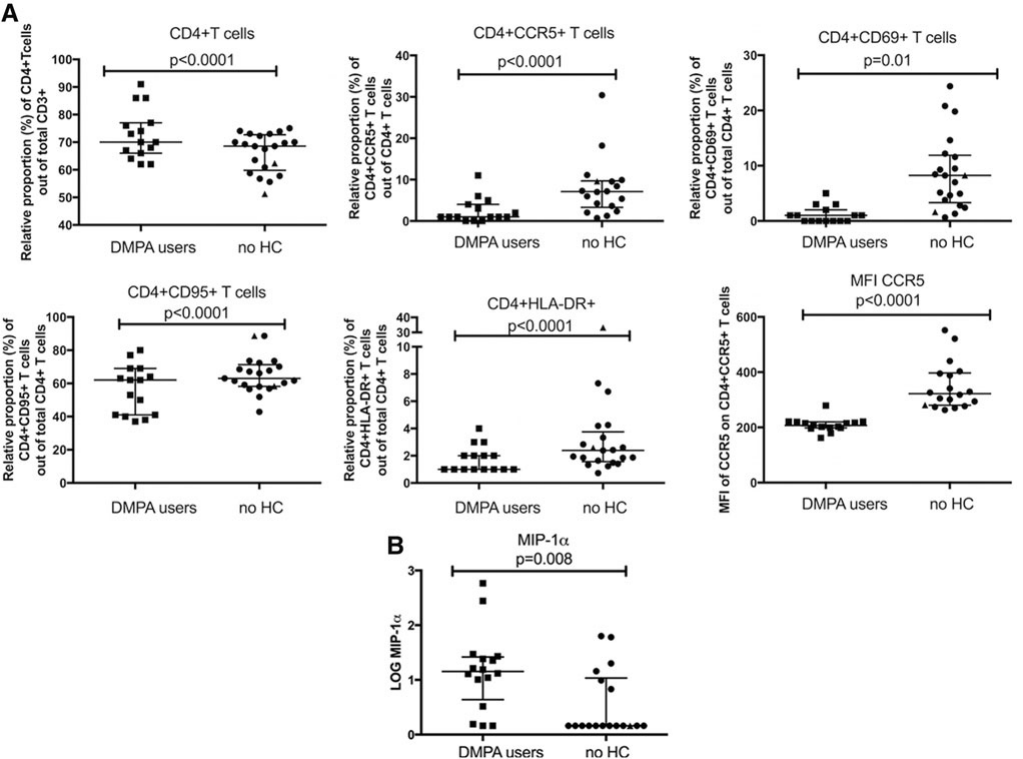
The impact of DMPA in blood samples. **(A)** PBMC expression of cellular markers of activation and HIV co-receptor assessed by flow cytometry; **(B)** Expression of proinflammatory cytokines and chemokines derived from plasma. *p*-Values are from the multivariate analyses; graphs are presented with median and interquartile range; “▴” indicate participants sexually transmitted infections positive. ▪, DMPA-users (*square*); •, no HC users (*circle*). DMPA, depot medroxyprogesterone acetate; HC, hormonal contraception; MFI, median fluorescence intensity; PBMC, peripheral blood mononuclear cells.

As duration of sex work and douching were different between our groups, we performed multivariate analyses to control for those two cofounding variables. All of these differences, except for CD4^+^CCR5^+^CD69^+^ T cells, remained significant after multivariate analysis (after adjustment: % CD4^+^ T cells among total CD3^+^ T cells, *p* < .0001; CD4^+^CCR5^+^ T cells, *p* < .0001; CD4^+^CD69^+^ T cells, *p* = .01; CD4^+^CD95^+^ T cells, *p* < .0001; and CD4^+^HLA-DR^+^ T cells, *p* < .0001 among the CD4^+^ T cell population; Fig. 1A).

Furthermore, univariate analysis (Supplementary Fig. S2A) of the median fluorescent intensity (MFI) of the selected markers revealed that the DMPA group, when compared with the no HC users, displayed significantly lower intensity of expression on a per cell basis of the HIV co-receptor CCR5 on the CD4^+^ T cells (median MFI: 207 vs. 322, *p* < .0001), but significantly higher levels of the activation markers CD69 (median MFI: 4872 vs. 2297, *p* < .0001) and CD95 (median MFI: 3959 vs. 2917, *p* = .02) on the CD4^+^ T cells. After adjusting for the co-founding variables “duration of sex work” and “douching” in the multivariate analysis, only the decreased expression intensity (MFI) of CCR5 on the CD4^+^ T cells remained significant between the groups (*p* < .0001; Fig. 1A). Collectively, women using DMPA had a lower portion of activated CD4^+^ cells in their blood compartment when compared with the no HC users.

### Impact of DMPA usage on plasma cytokines

Next, we addressed whether DMPA usage affected the levels of cytokines in blood. Thus, the levels of 19 selected cytokines/chemokines involved in inflammatory and immune activation pathways were measured in plasma samples and log10 transformed to normalize their distribution.

The univariate analysis (Supplementary Fig. S2B) showed that the DMPA group, relative to the no HC users, had significantly lower levels of IL-12p70 (median log_10_ transformed: −0.15 vs. 0.91; *p* = .04, and IL-1α (median log_10_ transformed: 0.67 vs. 0.67; *p* = .04), but significantly higher levels of MIP-1α (median log_10_ transformed: 1.15 vs. 0.16; *p* = .001). The level of IL-1β showed a trend to be higher in the DMPA users when compared with the no HC users (median log_10_ transformed: 0.15 vs. −0.4; *p* = .06). No differences were seen between the two groups for the other 15 cytokines analyzed (data not shown). After multivariate analysis (adjusting for “duration of sex work” and “douching”), only MIP-1α remained significantly different with the DMPA users having higher level compared with the no HC group (*p* = .008; Fig. 1B).

We next compared the number of participants that displayed cytokine levels over the limit of detection, and we observed that DMPA users were more likely to produce IL-1β (100% vs. 37% *p* = .001), MIP-1α (93% vs. 33% *p* = .002), and MIP-3α (100% vs. 88% *p* = .05) when compared with the no HC group (data not shown). In the multivariate analysis, only elevated levels of the proinflammatory cytokine MIP-1α among the DMPA group remained significant (data not shown).

### Impact of DMPA usage on cervical CD4^+^ T cells

To determine whether DMPA usage was associated with changes in the numbers of HIV target cells at the female reproductive tract, we assessed single cell suspensions of CMCs by flow cytometry as an internal quality control, CMC samples that had a count of less than 100 CD3^+^ in the flow cytometry “live gate” were excluded from further analyses. Univariate analysis (Supplementary Fig. S3A) showed that the proportion of cervical CD4^+^ T cells out of total cervical CD3^+^ T cells was similar between DMPA users versus no HC users (median: 52% vs. 48%, *p* > .05) and the proportion of CD4^+^CCR5^+^ cells among the cervical CD4^+^ T cell population (median: 28% vs. 28%, *p* > .05). However, the proportion of early activated/tissue residing cervical CD4^+^ T cells (CD4^+^CD69^+^) (median: 39% vs. 14%, *p* = 0.02), including HIV target cells (CD4^+^CCR5^+^CD69^+^) out of total cervical CD4^+^ T cells (39% vs. 9%, *p* = .006) was higher in DMPA versus no HC users. Moreover, while the expression level of CCR5 on cervical CD4^+^ T cells was significantly lower in the DMPA, when compared with the no HC users (median MFI: 308 vs. 497, *p* = .004), the expression level of CD69 on cervical CD4^+^ T cells was significantly higher in the DMPA compared with the no HC users (median MFI: 3349 vs. 1914, *p* = .002; Supplementary Fig. S3A).

Due to low cell numbers in some CMC preparations, the total number of study samples was lower in the CMC analyses compared to cell availability from blood in the same participants. One participant in the DMPA group and 13 in the no HC group were eliminated, as the CMC numbers did not reach our threshold, thereby resulting in an analyzed group size of 14 for DMPA and 6 to 8 for no HC. This necessitated a renewed analysis of sociodemographic differences between the study groups with sufficient CMC numbers for analysis. “Duration of sex work” remained significantly different between DMPA users and no HC users (median: 24 vs. 36 months, *p* = .03), while “douching” habits were now not statistically different (43% vs. 25%, *p* > .05) (data not shown). To remain consistent, we included both factors in the multivariate analyses.

**FIG. 2.**
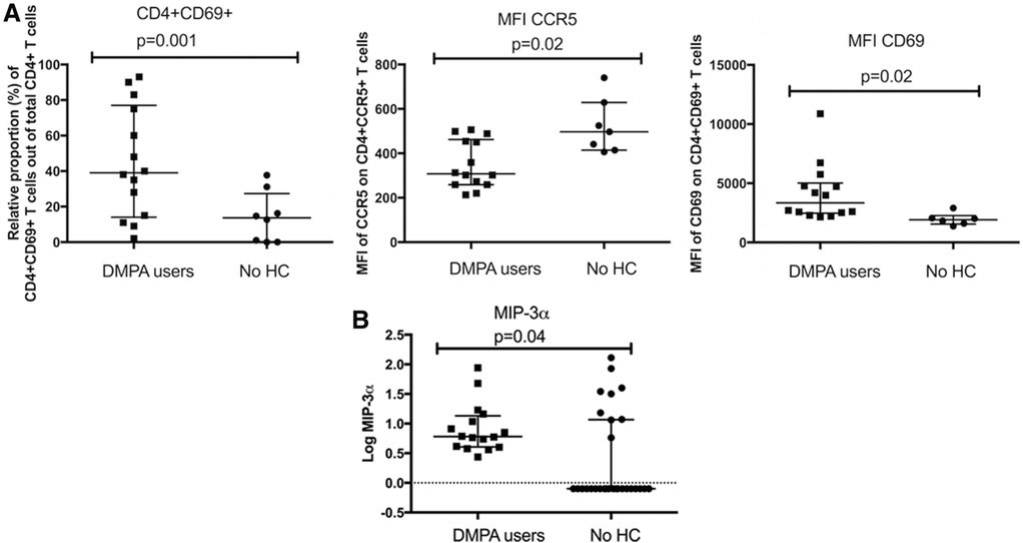
The impact of DMPA on mucosal milieu. **(A)** The impact of DMPA on mucosal CMCs. Expression of cellular markers of activation and HIV co-receptors on CMCs as assessed by flow cytometry. **(B)** The impact of DMPA on MIP-3α in CVL. Expression of the proinflammatory chemokine MIP-3α in the CVL. *p* -Values are from the multivariate analyses; graphs are presented as median and interquartile range. CMCs, cervical mononuclear cells; CVL, cervico-vaginal lavage.

This analysis showed the proportion of cervical CD4^+^CD69^+^ T cells among total cervical CD4^+^ T cells remained significantly higher in the DMPA group (*p* = .001), as did the expression levels of CCR5 and CD69 on cervical CD4^+^ T cells (*p* = .02 for both comparisons; Fig. 2A).

### Impact of DMPA on secreted cytokines in the female genital tract

To assess the proinflammatory environment, we then assessed the abundance of the selected cytokines/chemokines in CVL samples. Proteins found in CVL may originate from both the upper and lower female genital tract, and from plasma transudate, thus representing a broad picture of the immunological milieu within the female genital mucosa. Univariate analysis (Supplementary Fig. S3B) showed that the DMPA users, when compared with the no HC users, were characterized by higher level of IFN-γ (median log_10_ transformed: 0.16 vs. −0.40, *p* = .0001), IL-10 (−0.26 vs. −0.26, *p* < .0001), IL-17A (−0.004 vs. −0.46, *p* = .0002), IL-2 (−0.30 vs. −0.30, *p* = .035) MIP-3α (0.78 vs. −0.10, *p* = .003), and TNF-α (−0.11 vs. −0.46, *p* = 0.003) in their CVL samples. No differences were seen between the two groups for the other 13 cytokines analyzed. After adjusting for co-founding variables (“duration of sex work” and “douching”) in the multivariate analysis, only the level of MIP-3α remained significantly elevated in CVL of the DMPA group (*p* = .04; Fig. 2B).

When comparing the number of samples that displayed cytokines levels over the limit of detection, we observed that DMPA users, when compared with the no HC users, were more likely to produce high levels of IFN-γ (88% vs. 21%, *p* = .0001), IL-12p70 (81% vs. 32%, *p* = .006), IL-15 (44% vs. 11%, *p* = .0009), MCP-1 (100% vs. 68%, *p* = .02), and MIP-3α (100% vs. 32%, *p* < .0001), but not IL-17A (data not shown). In summary, MIP-3α was the only cytokine out of 19 tested that remained significantly increased when comparing the DMPA versus no HC groups following multivariate analysis.

### Impact of DMPA usage on HIV target cells in the ectocervical epithelium

**FIG. 3.**
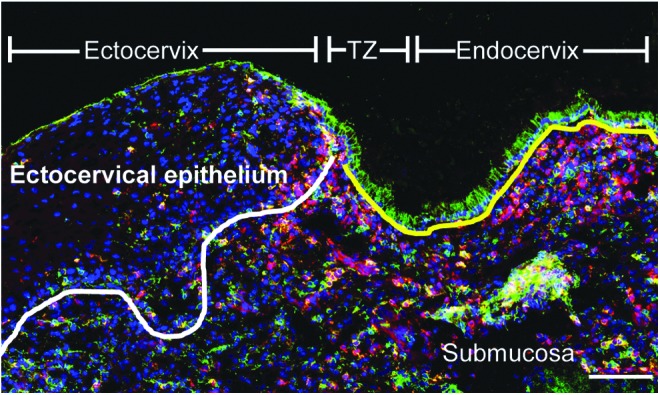
The transformation zone is highly populated with HIV target cells. Immunofluorescence images of cervical tissue sections from a DMPA user stained for CD4^+^ (*red*) and CCR5^+^ (*green*). DAPI (*blue*) was used as a counterstain for visualization of cell nuclei. The *white line* indicates the basal membrane, which separates the ectocervical epithelium from the underlying submucosal compartment, and the *yellow line* indicates the single columnar epithelium present in the endocervix. The *green* staining shown in the apical layer of the ectocervix and the endocervix is autofluorescent mucus. The scale bar represents 100 μm.

**FIG. 4.**
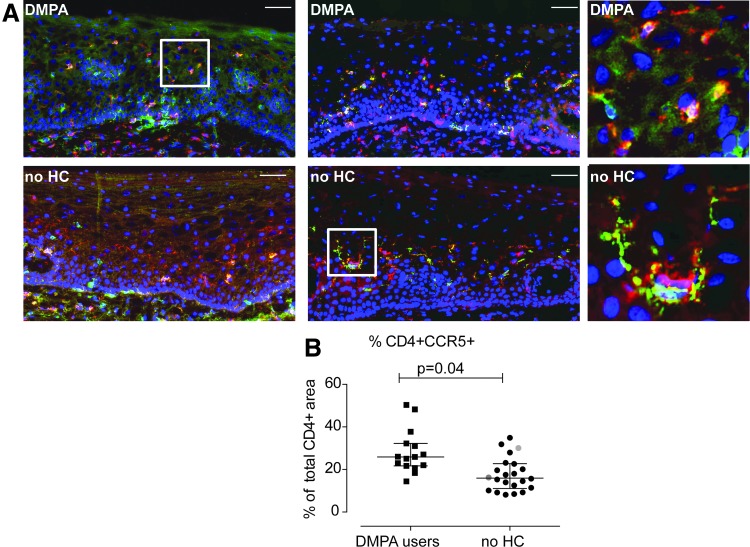
Visualization and enumeration of HIV target cells in the ectocervical epithelium of DMPA- and no HC users. **(A)** Representative immunofluorescence images of ectocervical tissue sections from a DMPA user (*upper row*) and a no HC user (*lower row*), stained for CD4^+^ (*red*) together with CCR5^+^ (*green*, *left column*), and CD4^+^ (*red*) together with Langerin^+^ (*green*, *middle column*); double-positive cells are shown in yellow. DAPI (*blue*) was used as a counterstain for visualization of cell nuclei. The images in the *right column* are magnified view of the regions indicated in the respective *box* in the images to the *left.* The scale bars represent 100 μm. **(B)** A scatter plot of percentage positively stained CD4^+^CCR5^+^ cell area out of total CD4^+^ stained tissue area. Each *square/circle* represents a different subject; DMPA-users (*square*) and no HC users (*circle*). As compared with the other analyses (PBMC, CMC, and CVL), two additional control subjects (no HC) were included and they are therefore clearly marked. One woman was infected with *Neisseria gonorrhoeae* (*green circle*) and one with *Chlamydia trachomatis* (*red circle*). These women were infected after enrollment and were therefore not excluded from entering the study. *Horizontal lines* represent median ± interquartile range. *p* values are from multivariate analyses.

In addition to comparing PBMC and plasma to genital tract CMCs and CVLs, this study had the advantage of also assessing ectocervical tissue samples for numbers of HIV target cells (Fig. 3). Thus, immunofluorescence staining of CD4 together with either CCR5 or Langerin was performed on ectocervical tissue sections (Fig. 4A). Computerized image analysis of the immunofluorescent double staining was performed and comparable ectocervical epithelial tissue areas were analyzed for both study groups, in both sets of staining (median areas for the CD4^+^CCR5^+^ staining: 6.9 × 10^5^ vs. 5.6 × 10^5^ μm^2^, *p* = .63; and median areas for the CD4^+^Langerin^+^ staining: 5.0 × 10^5^ vs. 5.1 × 10^5^ μm^2^, *p* = .66) (data not shown). The frequency of positively stained cells was here expressed as percentage of stained area, out of the total tissue area, and out of stained CD4^+^ tissue area.

Univariate analysis (Supplementary Fig. S4) showed no difference in the percentage of CD4^+^cells out of the total tissue area between the study groups in the CD4^+^CCR5^+^ staining (median: 3.0% vs. 3.0%, *p* = .65) and in the CD4^+^Langerin^+^ staining (median: 2.9% vs. 2.2% *p* = .15). However, the DPMA users, relative to the no HC users, displayed significantly higher percentage of CCR5^+^cells (median: 2.1% vs. 1.2%, *p* = .008), and Langerin^+^ cells (median: 2.0% vs. 1.4%, *p* = .04) out of the total tissue area. Moreover, the DMPA users, when compared with the no HC users, displayed a statistical trend of higher percentage of CD4^+^CCR5^+^ cells (median: 0.8% vs. 0.5%, *p* = .06) and a significantly higher percentage of CD4^+^Langerin^+^cells (median: 0.8% vs. 0.5%, *p* < .05) out of the total tissue area. Additionally, within the CD4^+^ cell pool, the proportion of CD4^+^CCR5^+^ cells was significantly higher in the cervical epithelium of DMPA users, when compared with the no HC users (median: 26% vs.16%, *p* = .002), while a similar proportion of CD4^+^Langerin^+^ cells was seen between the two study groups (median: 27% vs. 28%, *p* = .59). The shorter “duration of sex work” and more “frequent douching” recorded in the DMPA users versus no HC users were controlled for in a multivariate analysis. The proportion of CD4^+^CCR5^+^ cells among the CD4^+^ cell pool remained significantly increased in the DMPA user group (*p* = .04; Fig. 4B) whereas all other comparisons lost significance. Thus, these data indicate that the DMPA users displayed significantly higher levels of the main HIV target cells (i.e., CD4^+^CCR5^+^cells) in their ectocervical epithelium, when compared with the no HC users.

**FIG. 5.**
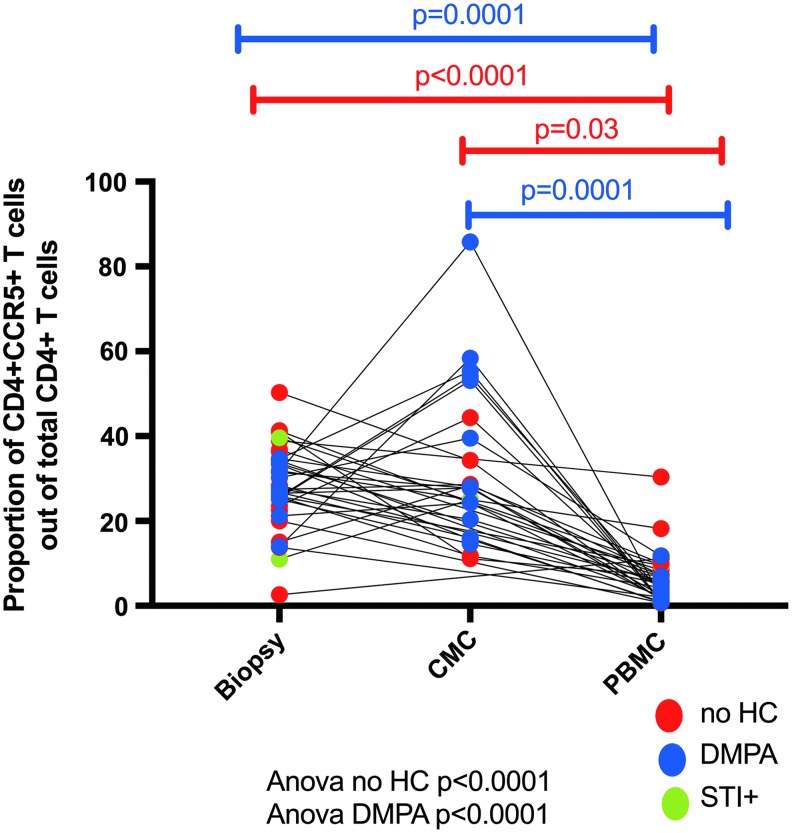
Proportion of CD4^+^CCR5^+^ T cells present in the blood, CMC, and biopsy (ectocervical tissue).

We compared the proportion of HIV target cells present between the three sites and observed a higher proportion of CD4^+^CCR5^+^ T cells at the genital compartment compared with the blood (Fig. 5).

## Discussion

DMPA has been epidemiologically associated with an increased risk of acquiring HIV.^3–5^ To determine the effect of DMPA in blood we analyzed PBMC and plasma. Following multivariate analysis, women using DMPA had a lower portion of activated CD4^+^ cells and elevated levels of the proinflammatory cytokine MIP-1α in the circulation, compared with the no HC user control group. It is interesting to note that the number of activated HIV target cells was actually lower in the blood of DMPA users suggesting a lack of their upregulation or their migration to other compartments.

Of greater relevance to the sexual transmission of HIV, was the effect of DMPA use on the female reproductive tract. Cervical CD4^+^ T cells of DMPA users had a higher proportion that expressed the early activation/tissue resident marker CD69 than no HC users. Furthermore, the intensity of expression of CD69, on a per cell basis of cervical CD4^+^ T cells was elevated in DMPA users. However, the intensity of expression of CCR5 on these cervical CD4^+^ T cells was lower in DMPA users. In ectocervical tissue samples, DMPA users had significantly higher numbers of CD4^+^CCR5^+^ T cells among the CD4^+^ T cell population in the epithelium when compared with no HC users. The observation that this elevation of HIV target cells was not seen among the CMC samples may indicate a compartmentalization of HIV target cells.

Important differences between the blood and mucosal compartments were observed. In blood, DMPA users had not only lower proportion of these HIV target cells, but also lower levels of activated T cells (CD4^+^CD69^+^, CD4^+^CD95^+^, and CD4^+^HLA-DR^+^) and expression levels of CCR5. In corresponding CMC samples, the CD4^+^CD69^+^ cell population was increased in DMPA users, and the expression intensity of CD69.

It has been shown that HIV infects activated T cells more efficiently than quiescent T cells. Therefore, the observation that DMPA users had higher proportion of CD69^+^ T cells is an important consideration for HIV susceptibility. As CD69 is considered a tissue residing marker, the higher density of CD69 on DMPA-users' CD4^+^ T cells in the CMC preparations also suggests a higher tissue retention of CD4^+^ T cells at this site. Indeed, CD69 is an early activation marker on T cells that also allows distinguishing between circulating and resident T cells.^29^ Therefore, by increasing the proportion of activated T cells and increasing the retention of those cells in the female genital tract, DMPA increases the pool of potential target cells for HIV at the portal of entry.

Another impact of DMPA was observed when comparing the expression of cytokines and chemokines in CVL. After multivariate analysis, MIP-3α was significantly elevated among CVLs of the DMPA users. MIP-3α is a chemokine that attracts lymphocytes and DCs into mucosal tissue near the epithelial barrier.^30,31^ It has been shown that the level of MIP-3α is rapidly increased after infection with HIV, which leads to the recruitment of plasmacytoid DCs and production of MIP-1α and MIP-1β, which in turn attracts activated CCR5^+^CD4^+^ T cells. Haase *et al.* have shown that by blocking this expression of MIP-3α, it is possible to prevent SIV infection in a simian model.^32,33^ Contrary to Francis *et al.*,^34^ we did not observe an increase in the levels of IL1-α, IL-1-β, MIP-1β, IP-10, and IL-8 in the DMPA user group, with only MIP-3α elevated in our study. This may suggest that the precise pattern of elevated proinflammatory cytokines correlated with DMPA use may be population dependent.

A unique feature of this study is the availability of matching ectocervical tissue biopsy samples to assess *in situ* cell distribution. Here, we use a novel computerized image analysis of immunofluorescent staining that was standardized in a rigorous manner to allow reliable quantification of phenotypic markers and location of HIV target cells in tissue samples.

The results were expressed as frequency of positively stained cells per tissue area and per stained CD4^+^ T cell tissue area. The total number of CD4^+^ cells per tissue area was comparable between the two study groups, whereas the number of total CCR5^+^ cells per tissue area was higher in the DMPA using group. Since CCR5 is also expressed on CD8^+^ T cells, we performed more detailed studies with double-staining techniques to better define which proportion of the CCR5^+^ cells were potential HIV target cells. It was thereby confirmed that the number of CD4^+^CCR5^+^ cells per tissue area trended to be higher for DMPA users, and that the CD4^+^CCR5^+^ cells out of stained CD4^+^ cell area was significantly higher in DMPA users. The latter finding remained significant when adjusted for the two confounders described above.

LCs,^35,36^ DCs,^37^ and T cells^33,38^ are early target cells in the female reproductive tract for SIV infection. More specifically, Th17-lineage CCR6^+^CD4^+^ T cells were identified as very early primary targets in a macaque SIV vaginal challenge model and infectious foci were identified throughout the reproductive tract.^39^

Studies in human vaginal explants^18^ suggest that HIV target cells in the genital tract mucosa not only include CD4^+^CCR5^+^ T cells, but also LCs. In addition to conventional binding to CD4 and CCR5; DCs, LCs, and macrophages can also bind HIV through other receptor pathways including CLRs. Abundant and superficial expression of CLRs have been found in ectocervix of women at risk of HIV infection: CD11c^−^CD1a^+^langerin^+^ cells were localized in the epithelium, whereas myeloid DCs and macrophages were restricted to the submucosa. Langerin (an LC-specific marker) was mainly expressed by intraepithelial CD1a^+^CD4^+^ and CD11c^+^CD4^+^ cells.^40^

In this study, women using DMPA expressed significantly increased numbers of CD4^+^CCR5^+^ cells *in situ* per total CD4^+^ tissue area when compared with women not using any form of hormonal contraception. However, the number of CD4^+^Langerin^+^ cells out of total CD4^+^ tissue area in the ectocervical epithelium was similar between the groups. Altogether, the results thus show that DMPA usage was associated with increased levels of the main HIV target cell population in the ectocervical epithelium of HIV-seronegative women sex workers.

There are several strengths of this study. As compared to many other studies, our study assessed the impact of DMPA in blood and in the mucosal compartment. Furthermore, at the mucosal compartment we compared the cell populations in both CMCs and *in situ* in ectocervical tissue samples. The criteria used to define the luteal phase for our control group was stringent and represents the natural high level progesterone phase to most closely mimic the impact of the high-progestin contraceptive DMPA. Furthermore, only women with long-term DMPA use and a recent DMPA injection were included to have a stringently defined study group. As DMPA was previously associated with increased risk of HIV infection, understanding how it impacts the immune response in women who are actually at high risk of HIV infection is highly relevant to the field. Therefore, we consider that the enrolment of FSWs in an HIV-endemic area is an additional strength of the study.

There are also some important limitations that need to be accounted for when interpreting this study. These include the limited sample size, especially for the CMC analysis, and the differences in two of the demographic parameters (duration of sex work and frequency of douching). By performing multi-variate analyses, we controlled for these two important confounding variables. CMC samples may contain semen-derived contaminants and the sampling procedure as such may introduce blood-borne leukocytes into resident cell samples.^41^ However, visible red blood cells in cytobrushes correlated with increased leukocyte yields but these were largely derived from the mucosa and not peripheral blood as suggested from their subpopulation profile. Furthermore, the tissue sample collection procedure including sampling of small surface areas may not fully represent the areas most susceptible to HIV.^41^

In this study, DMPA users were more likely to perform vaginal douching. Our questionnaire does not provide us details about this behavioral difference but future studies should carefully look at this practice as it affects the mucosal milieu.

In this study, we showed that DMPA differentially affects the systemic and mucosal compartment relative to women who do not use hormonal contraceptives. The DMPA users had more activated CD4^+^ T cells in the CMC population and a higher proportion of CD4^+^CCR5^+^ T cells in the ectocervical epithelium, which faces the vaginal lumen. The increased pool of potential HIV-target cells and activated T cells provide a potential biological basis for the epidemiological association between DMPA use and HIV acquisition risk. Our study highlights the importance of conducting extended studies on the impact of DMPA in high-risk populations such as sex workers.

## Supplementary Material

Supplemental data

Supplemental data

Supplemental data

Supplemental data
