# Supplementary material for: Increased Cervical CD4+CCR5+ T Cells Among Kenyan Sex Working Women Using Depot Medroxyprogesterone Acetate
Source: AIDS Res Hum Retroviruses. 2019 Feb 28;35(3):236–46. doi: 10.1089/aid.2018.0188 (PMC6434599; doi:10.1089/aid.2018.0188)
Supplement: Supplemental data [file Supp_Fig1.pdf]

# Supplementary Data

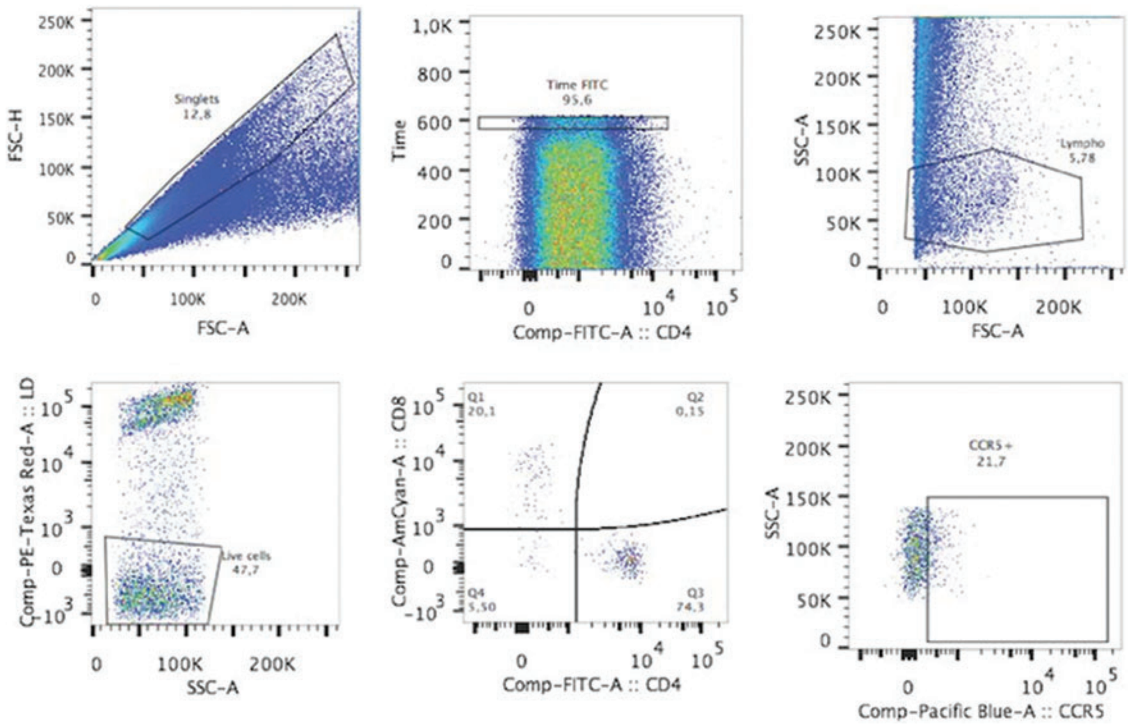


**SUPPLEMENTARY FIG. S1.** Gating strategy for flow cytometry analysis of cervical mononuclear cells. Freshly isolated CMCs were stained by the cocktails of fluorescently labeled antibodies described in the Materials and Methods section. Cells were first gated on singlet cells, then time of acquisition was determined to ensure it was continuous. Lymphocytes were gated by forward and side scatter profiles and viability determined by far-Red-live dead/SSC-A gating. $CD3^+$ lymphocytes were gated into $CD4^+$ or $CD8^+$ populations and surface markers such as CCR5 determined related to a fluorescence-minus-one negative gate. CMCs, cervical mononuclear cells; FSC-A, forward scatter area; FSC-H, forward scatter height; SSC-A, side scatter area.
