# Supplementary material for: Increased Cervical CD4+CCR5+ T Cells Among Kenyan Sex Working Women Using Depot Medroxyprogesterone Acetate
Source: AIDS Res Hum Retroviruses. 2019 Feb 28;35(3):236–46. doi: 10.1089/aid.2018.0188 (PMC6434599; doi:10.1089/aid.2018.0188)
Supplement: Supplemental data [file Supp_Fig2.pdf]

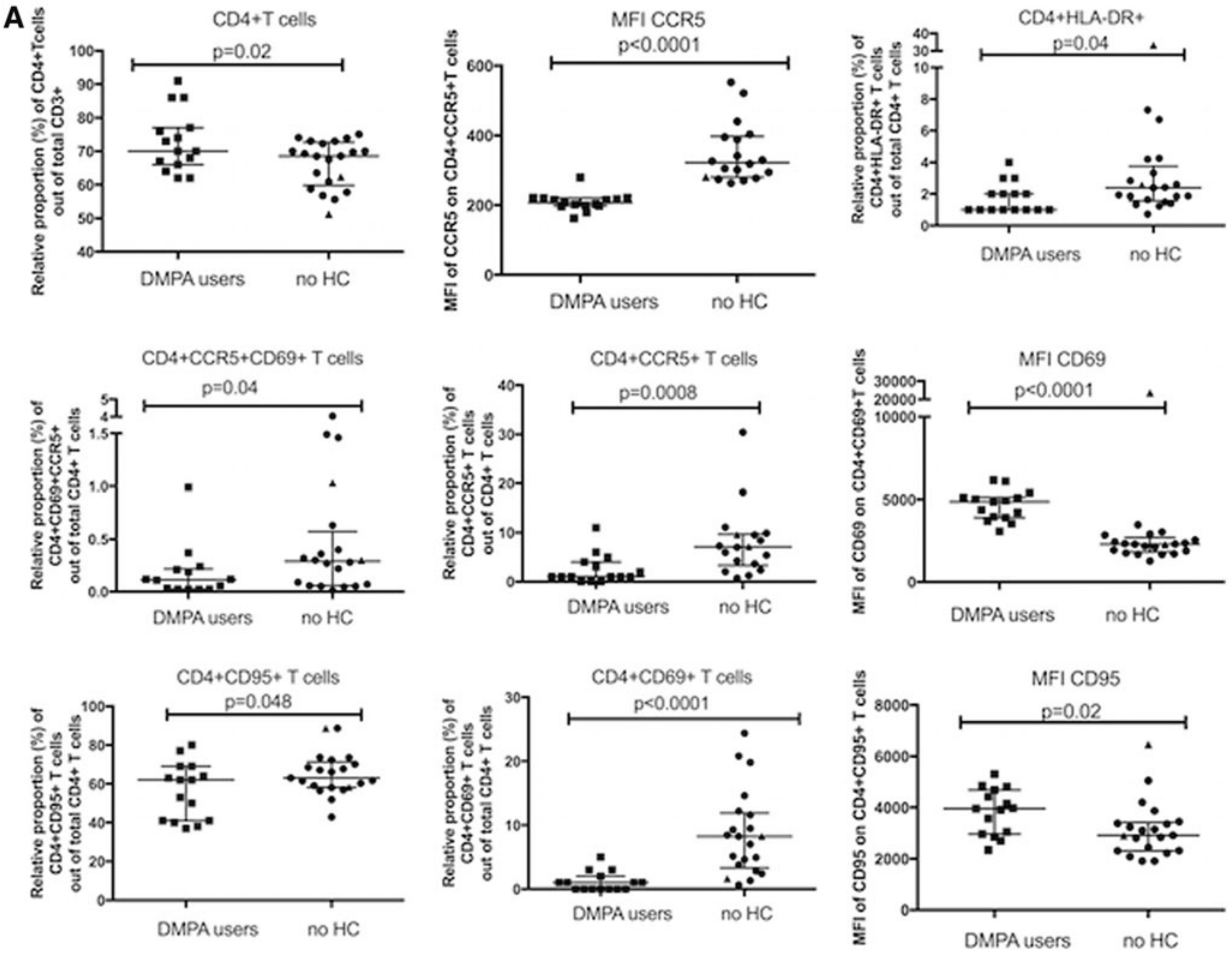

SUPPLEMENTARY FIG. S2. The impact of DMPA on the systemic immune system after univariate analyses. **(A)** PBMC expression of cellular markers of activation and HIV co-receptor assessed by flow cytometry; **(B)** Expression of proinflammatory cytokines and chemokines derived from plasma. *p*-Values are from the univariate analyses; graphs are presented with median and interquartile range; ''▲'' indicate participants STIs positive. ■, DMPA users (square); ●, no HC (circle). DMPA, depot medroxyprogesterone acetate; HC, hormonal contraception; MFI, median fluorescence intensity; PBMC, peripheral blood mononuclear cells; STI, sexually transmitted infection.

(*continued*)

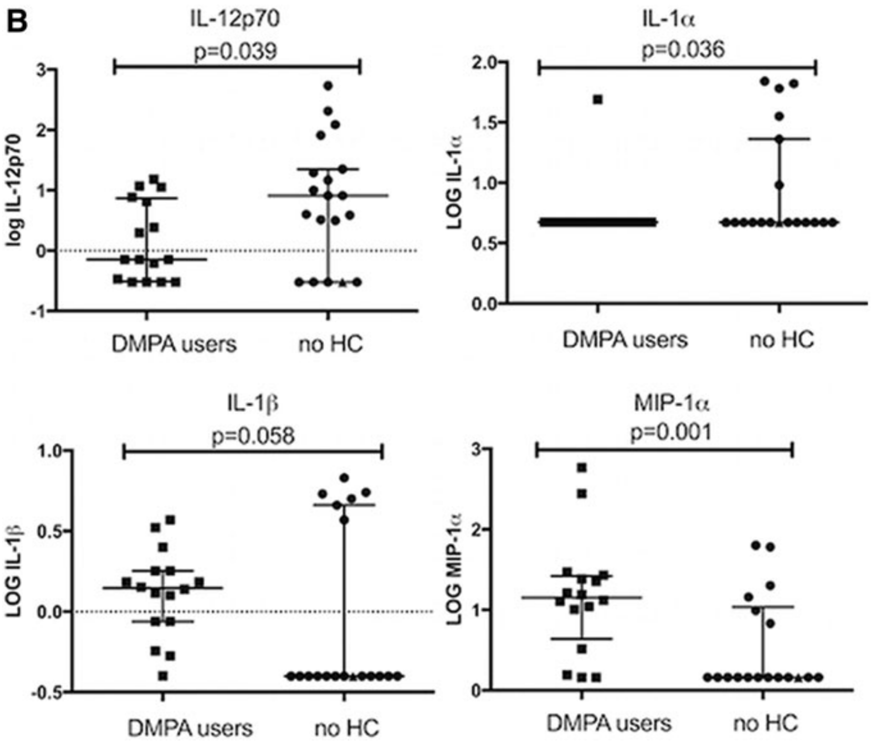

SUPPLEMENTARY FIG. S2. (Continued).
