# Supplementary material for: Increased Cervical CD4+CCR5+ T Cells Among Kenyan Sex Working Women Using Depot Medroxyprogesterone Acetate
Source: AIDS Res Hum Retroviruses. 2019 Feb 28;35(3):236–46. doi: 10.1089/aid.2018.0188 (PMC6434599; doi:10.1089/aid.2018.0188)
Supplement: Supplemental data [file Supp_Fig3.pdf]

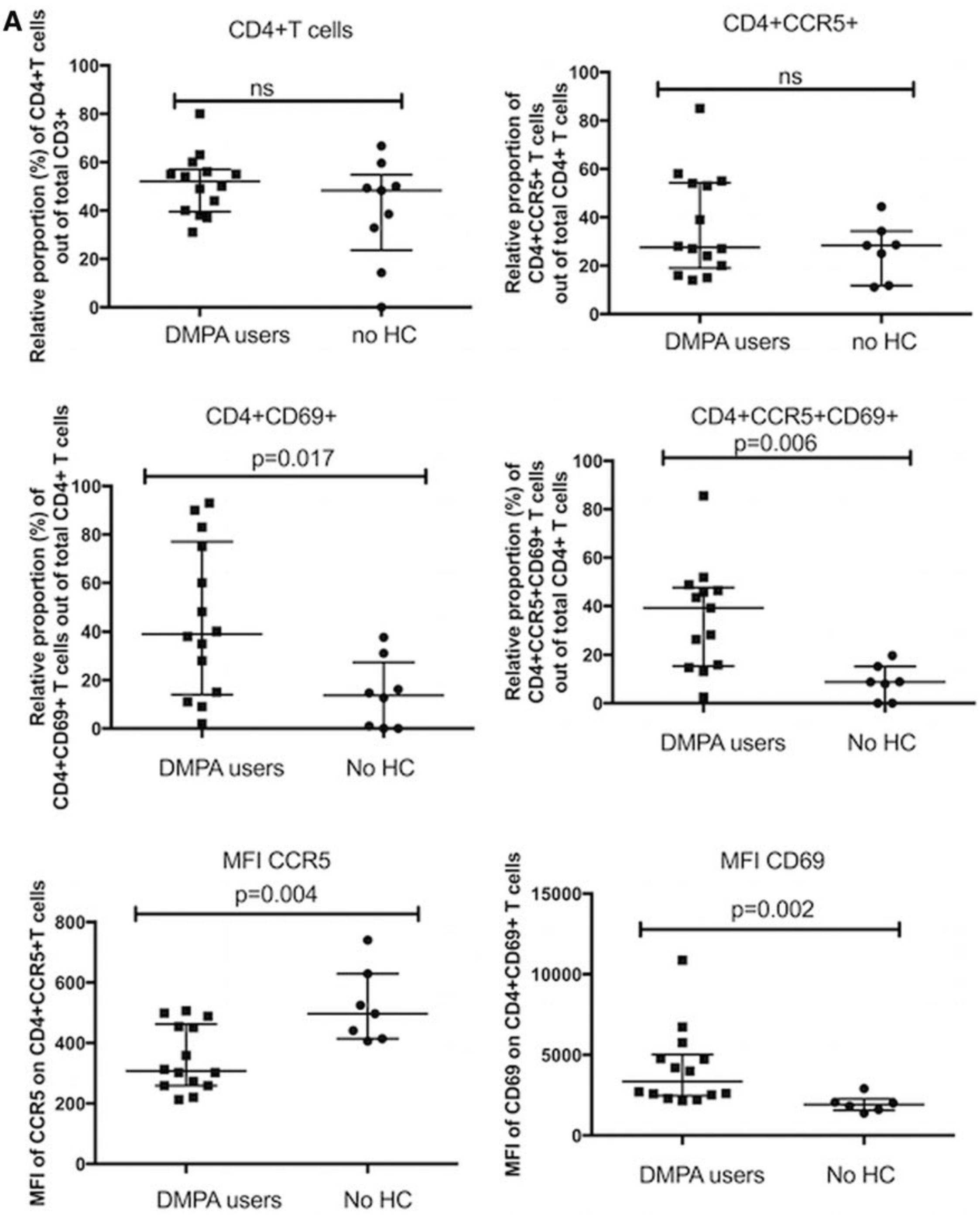

SUPPLEMENTARY FIG. S3. The impact of DMPA on mucosal milieu after univariate analyses. **(A)** Expression of cellular markers of activation and HIV co-receptors on CMCs as assessed by flow cytometry. **(B)** Expression of the proinflammatory chemokine in the CVL. *p*-Values are from the univariate analyses; graphs are presented as median and interquartile range. CVL, cervico-vaginal lavage.

(*continued*)

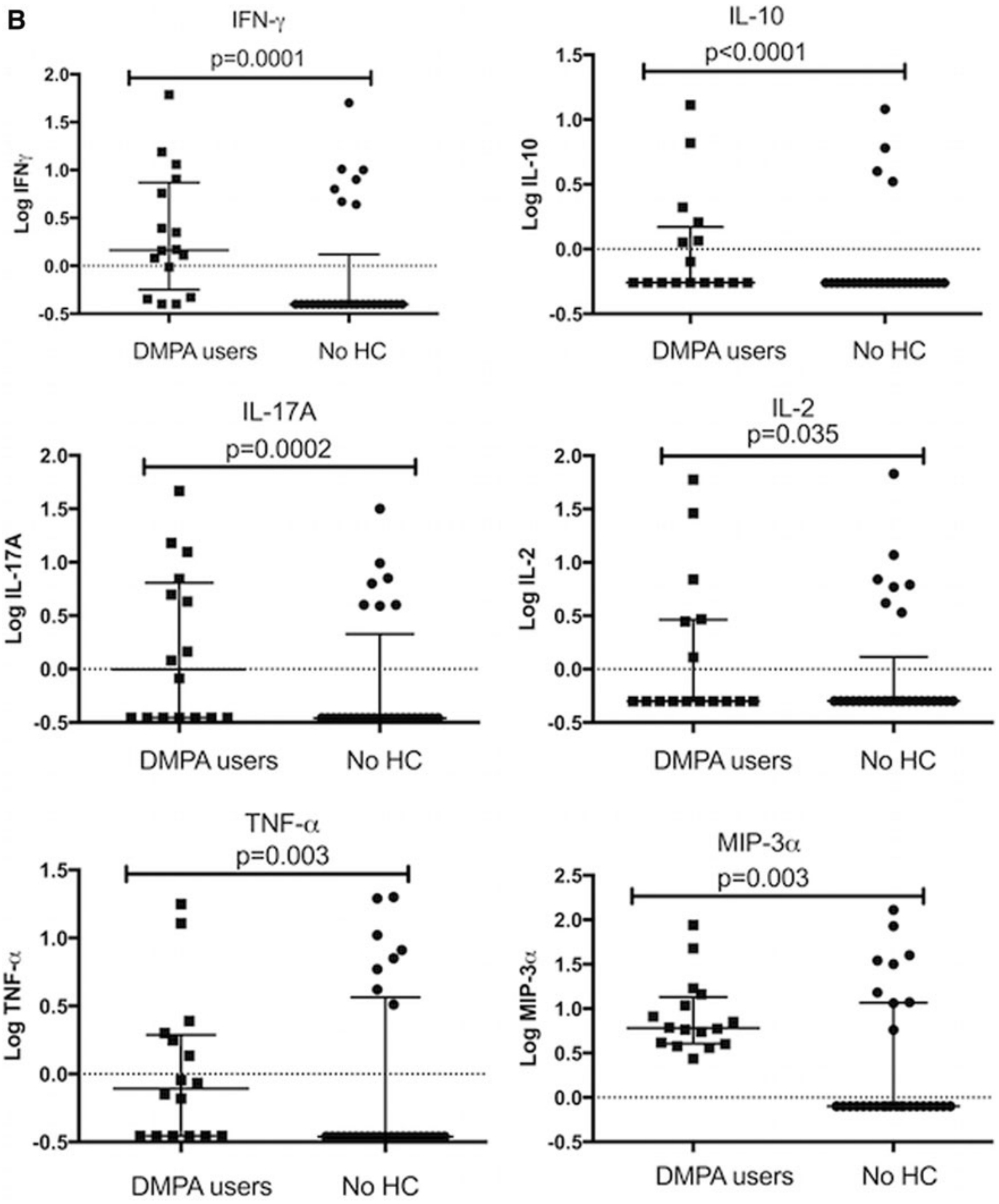

SUPPLEMENTARY FIG. S3. (Continued).
