# Supplementary material for: Increased Cervical CD4+CCR5+ T Cells Among Kenyan Sex Working Women Using Depot Medroxyprogesterone Acetate
Source: AIDS Res Hum Retroviruses. 2019 Feb 28;35(3):236–46. doi: 10.1089/aid.2018.0188 (PMC6434599; doi:10.1089/aid.2018.0188)
Supplement: Supplemental data [file Supp_Fig4.pdf]

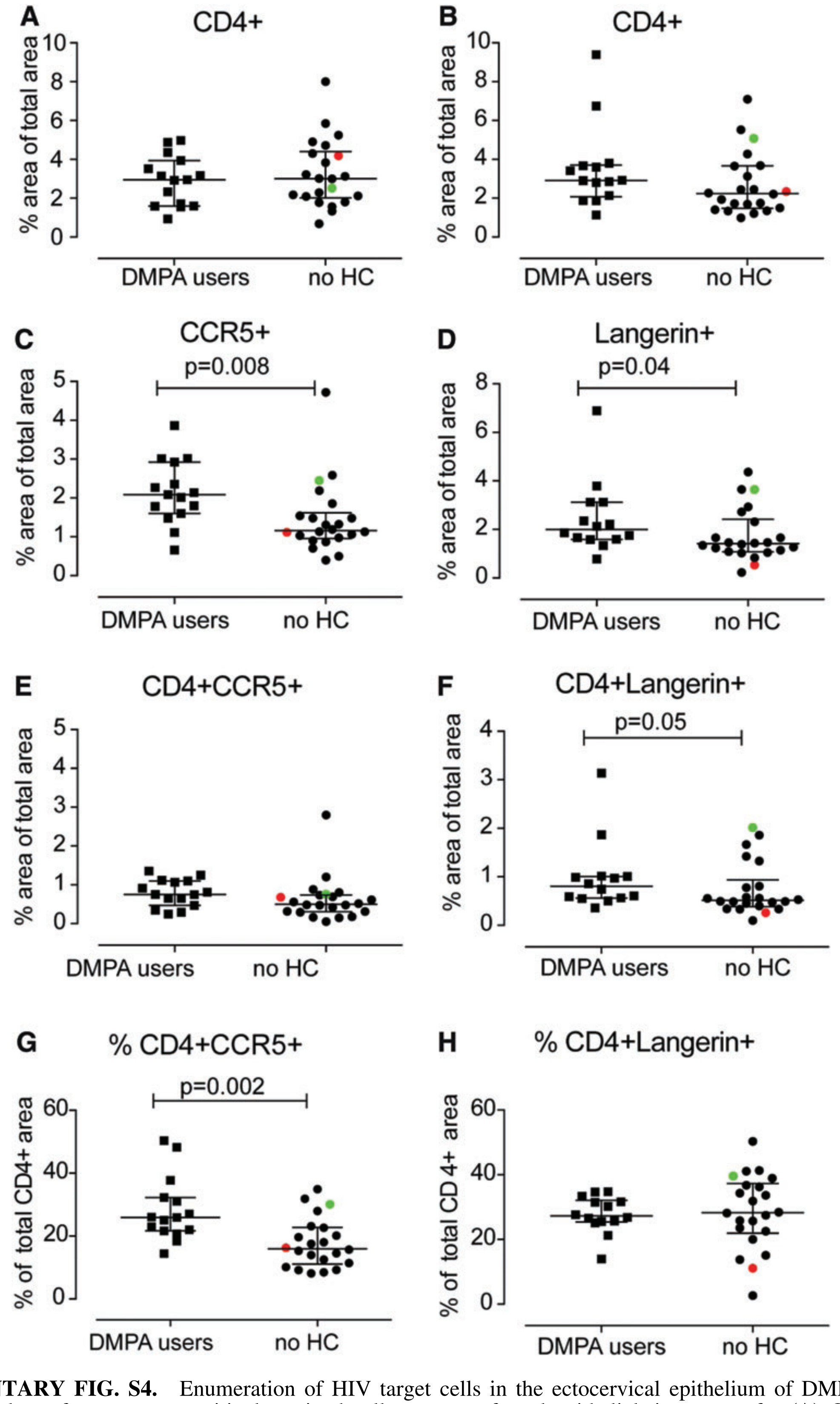

SUPPLEMENTARY FIG. S4. Enumeration of HIV target cells in the ectocervical epithelium of DMPA and no HC users. Scatter plots of percentage positively stained cell area out of total epithelial tissue area for **(A)** $CD4^+$ (from the $CD4^+CCR5^+$ staining, **(B)** $CD4^+$ (from the $CD4^+Langerin^+$ staining), **(C)** $CCR5^+$, **(D)** $Langerin^+$, **(E)** $CD4^+CCR5^+$, **(F)** $CD4^+Langerin^+$ cells, and **(G)** $CD4^+CCR5^+$ area out of total $CD4^+$ stained area and, **(H)** scatter plots of percentage positively stained $CD4^+Langerin^+$ area out of total $CD4^+$ stained area. Each *square/circle* represents a different subject; DMPA-users (*square*) and no HC users (*circle*). As compared with the other analyses (PBMC, CMC, CVL), two additional control subjects (no HC) were included and they are therefore clearly marked. One woman was infected with *Neisseria gonorrhoeae* (*green circle*) and one with *Chlamydia trachomatis* (*red circle*). These women were infected after enrollment and were therefore not excluded from entering the study. *Horizontal lines* represent median ± interquartile range. *p*-values are from univariate analyses.
